# What does Williams syndrome reveal about the determinants of social behavior?

**DOI:** 10.3389/fnhum.2013.00321

**Published:** 2013-06-28

**Authors:** Anna M. Järvinen, Ursula Bellugi

**Affiliations:** Laboratory for Cognitive Neuroscience, The Salk Institute for Biological StudiesLa Jolla, CA, USA

**Keywords:** Williams syndrome, social motivation, social behavior, autonomic nervous system, heart rate, oxytocin, arginine vasopressin

## Abstract

Growing evidence on autonomic nervous system (ANS) function in individuals with Williams syndrome (WS) has begun to highlight aberrancies that may have important implications for the social profile characterized by enhanced social motivation and approach. In parallel, neurobiological investigations have identified alterations in the structure, function, and connectivity of the amygdala, as well as prosocial neuropeptide dysregulation, as some of the key neurogenetic features of WS. A recent social approach/withdrawal hypothesis (Kemp and Guastella, 2011) suggests that autonomic cardiac control may play a key role in regulating the relationship between oxytocin (OT) and social behavior. This article discusses evidence from these critical, new strands of research into social behavior in WS, to consider the extent to which data on WS may provide novel insight into the determinants of social behavior. Future research directions are suggested.

## Introduction

Elucidating the origins of human social behavior has relevance to both typical and atypical development. In this vein, the unusual social phenotype of Williams syndrome (WS) has been gaining momentum among the neuroscience community. WS provides an attractive model for social/cognitive neuroscience because the hemideletion of 25–28 genes on chromosome 7q11.23 is well-characterized (Korenberg et al., 2000). Further, the phenotype comprising distinct socially positive and dysfunctional behaviors that implicate several neural systems is observed with remarkable consistency. The neurocognitive profile of WS is associated with mean IQ of 50–60, with typically higher verbal than non-verbal abilities (Searcy et al., 2004; Mervis and John, 2010).

The unusual social behavior of WS spans three discrete dimensions: enhanced motivational social drive, atypical emotional sensitivity, and increased salience of social stimuli (Järvinen et al., 2013). Social limitations are underscored by paradoxes suggesting that although such individuals keenly instigate social engagement they lack the skill to sustain a conversation and make friendships (Davies et al., 1998), and while they seem socially uninhibited they suffer from diagnostically significant non-social anxiety, attentional problems, and social maladjustment (Davies et al., 1998; Leyfer et al., 2006). In short, the genetically determined expression of hypersociability of WS combines with inadequate tools and skills to navigate and act appropriately in the social world. The profile of WS raises several fascinating questions regarding the underpinnings of the enhanced social drive.

There has been a recent expansion of research into the social brain in WS (e.g., Haas and Reiss, 2012; Järvinen et al., 2013). This body of work has indicated alterations in the structure and function of the amygdala, fusiform face area (FFA), and insula. In addition, atypical connectivity between the amygdala and the FFA, the orbital-frontal regions, and the insula, as well as within the frontostriatal pathway, has been reported. At the same time, the role of the autonomic nervous system (ANS) function remains an overwhelmingly under-researched area among researchers addressing the social profile of WS. The link between the amygdala, ANS function, and subsequent social behavior is a significant one: the amygdala is critically involved in both appetitive and aversive affective processing (Aggleton, 2000) and in emotional evaluation that contributes to social behavior (Adolphs, 2009). The amygdala further mediates affective arousal (LeDoux, 2000; Laine et al., 2009), and direct amygdala stimulation results in a robust skin conductance response (SCR) in humans (Mangina and Beuzeron-Mangina, 1996). As evidence implicates aberrancies in both the amygdala (e.g., Meyer-Lindenberg et al., 2005; Haas et al., 2009; Haas and Reiss, 2012) and ANS responsivity (e.g., Doherty-Sneddon et al., 2009; Plesa Skwerer et al., 2009; Järvinen et al., 2012; Riby et al., 2012a) in WS, the aim of this mini-review is to examine the extent to which ANS function may contribute to the characteristic social behavior of WS. We begin by briefly discussing the role of the ANS function and its regulation by prosocial neuropeptides in social–emotional behavior generally, followed by a review of the relevant literature on WS. We will discuss how the landmark social characteristics of WS converge with the ANS features, to determine the extent to which WS may offer insight into the origins of social behavior.

## ANS function and social behavior

The postulated relationship between sociability and ANS function reflects an old idea: for example, in the 1960s, Eysenck hypothesized that individual differences in the cortical processing of arousal are linked to emotional experience and social behavior. Specifically, whereas extraverted individuals are characterized by chronic under-arousal, which leads them to actively seek out stimulation (e.g., social engagement), introverted individuals display the opposite pattern of both ANS arousal and subsequent behavior (Eysenck, 1967, 1994, 1997, but see Beauducel et al., 2006). Thus, relative to introverts, extraverts have been described as inherently less aroused and arousable (Stelmack, 1990; Smith, 1994); exhibit decreased heart rate (HR) reactivity (Smith et al., 1995); lower skin conductance levels (SCL) (Smith et al., 1986); reduced phasic SCR (Smith et al., 1990); and faster electrodermal habituation (Smith et al., 1995).

Honing in on the role of the ANS in human sociability, the polyvagal theory (Porges, 2003, 2007; Porges and Furman, 2011) posits that specifically autonomic cardiac control is critically implicated in social behavior and attachment. An evolutionarily important dynamic regulatory system enables adaptive responses: when under threat, the “vagal brake” is released reflecting survival-promoting energy consumption. In contrast, the ANS promotes positive approach-related behaviors during secure times. The neural circuit known as the social engagement system, which is under cortical regulation, comprises a key component of the social ANS (Porges, 2007). The heart is innervated in a dual fashion by both the sympathetic and parasympathetic branches of the ANS, with an acceleration in HR being linked to greater sympathetic influence, and a decrease to greater parasympathetic involvement. Consequently, HR variability (HRV) is regarded as a direct index of parasympathetic NS activity (Bernston et al., 2005). Indeed, it has been hypothesized that resting state HRV is a biomarker reflecting an individual's capacity for approach-related motivations for social interaction (Kemp et al., 2012a,b; Patriquin et al., 2013). For example, autism is associated with decreased HRV (Bal et al., 2010), and higher baseline HRV amplitudes have been linked to improved social behavior and receptive language abilities in such individuals (Patriquin et al., 2013). The link between social–emotional behavior and autonomic cardiac control is thought to lie in the abundant connectivity between brain regions modulating ANS activity and emotion perception (Smith and DeVito, 1984; Thayer et al., 2009). Indeed, this psychophysiological biomarker is a useful research tool since the key aspect of social behavior, the motivation to approach or withdraw, may not always be overt and observable (Kemp et al., 2012b).

A further rationale for focusing on the ANS function in WS in an attempt to illuminate the underpinnings of its unusual social–emotional behavior comes from a recent study implicating the endogeneous dysregulation of prosocial neuropeptides, oxytocin (OT), and arginine vasopressin (AVP), in the social phenotype of WS (Dai et al., 2012). More specifically, this investigation reported increased baseline OT levels together with increased OT and AVP responses to emotional stimulation, in individuals with WS contrasted with typical controls (Dai et al., 2012). A contrasting profile is reported in autism, characterized by low plasma OT levels (Modahl et al., 1998). These hormones are proposed to play a key role not only in transient social behaviors but also in broader states and orientations, such as anxiety, social motivation, and the salience of social stimuli (Churchland and Winkielman, 2012). The association between ANS function and social behavior is underscored by recent evidence suggesting the mediating effect of OT. Specifically, according to a recent social approach/avoidance hypothesis (Kemp and Guastella, 2011; Quintana et al., 2013), OT increases social approach behaviors and may either be adaptive or maladaptive. The paraventricular and optical nuclei of the hypothalamus are responsible for the synthesis of OT, with direct OT projections to the dorsal brain stem, which is vital for cardiac regulation (Buijs et al., 1978). OT receptors are widespread in the central and peripheral nervous system (NS), with pronounced concentrations in brain regions critically implicated in complex social behaviors (Landgraf and Neumann, 2004). Neuroimaging data pinpoint contingencies between the effects of OT and the nature of the stimulus: OT decreases amygdala responses for fearful faces, while increasing responses for happy faces (Gamer et al., 2010). Autonomic control may also be mediated by OT via its actions on the amygdala, which expresses OT receptors in high density (Tribollet et al., 1992), and mediates intricate ANS responses (Davis and Whalen, 2001). The theory of Kemp and Guastella (2011) is ultimately congruent with the polyvagal theory (Porges, 2007): increased HRV following extraneous OT administration is observed (Kemp et al., 2012a,b), and the socially withdrawn predisposition of autism is associated with reduced HRV (Kemp et al., 2010). Animal studies have also suggested the link between OT and HRV (Grippo et al., 2009). Further support to the link between ANS function and OT is provided by findings suggesting that intranasal OT administration elicits pupil dilation, which has been suggested to promote approach behaviors (Wiseman and Watt, 2010). The exact mechanism via which OT influences central brain structures implicated in autonomic cardiac control or social cognition is currently poorly understood (Quintana et al., 2013). However, as new evidence may suggest alterations in social reward, social salience, and social motivational functions in WS (Dai et al., 2012), in light of the above literature, the ANS emerges as an attractive candidate for aspects of the altered social–emotional behaviors associated with WS.

## Linking social behavior with ANS function in WS

WS is characterized by a robustly established increased appetitive drive toward social interaction (see Järvinen-Pasley et al., 2008, for a review). Hallmark features of this characteristic include an unusually gregarious, friendly, un-shy, and people-oriented personality (Klein-Tasman and Mervis, 2003), increased attraction specifically toward unfamiliar people (Bellugi et al., 1999; Doyle et al., 2004), and a bias toward viewing faces and eyes (Mervis et al., 2003; Riby and Hancock, 2008). Thus, social information appears atypically salient for individuals with WS, manifesting as an attentional bias toward social over non-social stimuli (e.g., Järvinen-Pasley et al., 2008; Riby and Hancock, 2009a,b), as well as more competent cognitive processing of social than non-social stimuli (Järvinen-Pasley et al., 2010). Taken at face value, these behavioral features may implicate ANS responsivity patterns in WS that correspond to the extraverted personality profile, increased HRV, and elevated plasma levels of OT, indexing increased approach-related motivation and heightened salience of social stimuli (Eysenck, 1967; Porges, 2007; Kemp and Guastella, 2011). As will become apparent below, studies addressing HR and/or electrodermal activity (EDA) in WS are sparse, and have produced mixed results. The aim of the section below is to determine the extent to which the social behavioral profile of WS appears in tune with what is known about the underlying ANS function in the syndrome.

### EDA-based findings on ANS function in WS

Initial evidence suggested reduced autonomic arousal to face stimuli in individuals with WS (Plesa Skwerer et al., 2009). In this study, participants with WS, CA-matched TD controls and those with intellectual disabilities with were presented with dynamic faces expressing anger, disgust, fear, happiness, sadness, surprise, and neutral expression, while SCR and HR were monitored (Plesa Skwerer et al., 2009). However, as a control condition included neutral nature scenes, SCRs to social stimuli were increased relative to the non-social stimuli. Moreover, a subsequent study hinted that the finding that suggested hypoarousal to faces in WS may reflect the artificial nature of the face stimuli: the stimuli used by Riby et al. (2012a) incorporated both live and video-mediated displays of happy, sad, and neutral faces. Results showed that while video-mediated faces failed to increase the SCL in individuals with WS, live faces elicited the typically observed increase in arousal. Further, lower than typical SCLs were reported in participants with WS, which were interpreted as reflecting general hypoarousal in WS. Doherty-Sneddon et al. (2009) measured changes in SCR in individuals with WS and CA-matched TD controls during arithmetic tasks varying in both complexity and the degree of eye contact with the experimenter. Another task assessed the degree of gaze aversion related to cognitive load. The results indicated that while individuals with WS showed general hypoarousal and reduced gaze aversion in the naturalistic, live social interaction context, similar to the TD controls, their arousal levels elevated in response to face stimuli. This led Doherty-Sneddon et al. (2009) to suggest that atypically low general arousal level (Plesa Skwerer et al., 2009; Riby et al., 2012a) may underlie the tendency of individuals with WS to hold gaze for extended periods. At the same time, eye contact during cognitive processing leads to the typical decline in performance also in individuals with WS (Riby et al., 2012b), suggesting that holding direct gaze is taxing for such individuals as well. The finding of general hypoarousal in WS indeed appears consistent with that linked to the extraverted personality profile (Eysenck, 1967), as is that of reduced SCRs to social stimuli (Plesa Skwerer et al., 2009). The only significant EDA-related finding reported by Järvinen et al. (2012) showed a lack of typical habituation to faces in individuals with WS, indexing increased novelty value of face stimuli. In the visual component of the study, adults with WS and CA-matched TD individuals were presented with static images of happy, fearful, and neutral faces and non-social scenes. The authors suggested that the absence of habituation to faces may provide an ANS correlate for the increased interest in face stimuli observed in WS, as faces may appear atypically novel and original despite the repeated exposure in everyday life. This feature may thus contribute to the increased approach-related motivation in WS.

### Cardiac-based findings on ANS function in WS

Plesa Skwerer et al. (2009) reported increased interest in faces in individuals with WS, on the basis of findings of increased HR deceleration to such stimuli. This finding is consistent with the WS social profile. By contrast, utilizing more complex HR-derived analyses than those in the previous studies, Järvinen et al. (2012) found a general acceleration in mean HR for face stimuli in individuals with WS as compared to TD controls, together with decreased HRV to such stimuli. These results suggest increased emotional reactivity to the affective face stimuli in WS, as vagal control was diminished for social–affective information. This ANS profile is in fact in line with that associated with social anxiety (Elsesser et al., 2006; Wieser et al., 2009). This is surprising in light of findings that WS is specifically associated with anxiety that is non-social in nature (Leyfer et al., 2006). At the same time, approach-related motivation is also associated with increased autonomic arousal (Pönkänen and Hietanen, 2012). In the auditory modality, happy, fearful, and sad vocal relative to musical emotional stimuli elicited increased HRV in participants with WS only, suggesting reduced arousal to auditory social information. This pattern is in contrast to that reported in the visual domain. Additionally, WS was characterized by greater HRV as compared to the TD controls. Järvinen et al. (2012) interpreted the results to suggest that human vocalizations appeared more engaging than the music stimuli for individuals with WS, as HR deceleration reflects increased focused attention. Across the visual and auditory modalities, WS was further associated with elevated HRV to happy stimuli. This result indexing greater vagal involvement is in line with the positive bias frequently documented in individuals with WS (Dodd and Porter, 2010), as positively valenced emotional stimuli are specifically socially engaging promoting approach-related motivations (Porges, 2007).

### Pupil dilation as an index of ANS activity in WS

Studies quantifying pupil dilation in WS have reported attenuated pupil dilation in response to social stimuli in such individuals relative to CA and mental age (MA) matched TD participants, suggesting decreased ANS arousal to social information (Plesa Skwerer et al., 2011). In this study, participants were presented with social and non-social images, and notably, all groups exhibited increased arousal to the social as compared to non-social visual stimuli. The participants with WS also showed reduced pupil dilation to negative facial expressions as compared to controls. This finding is consistent with both behavioral and neurobiological reports indicating insensitivity to negative social information in individuals with WS (Meyer-Lindenberg et al., 2005; Haas et al., 2009; Santos et al., 2010), a feature that is thought to contribute to the increased affiliation with unfamiliar people in WS. Taken together, the ANS findings suggest a complex pattern of ANS function indexed by EDA, cardiovascular reactivity, and pupil dilation, underpinning the social profile of WS.

### Prosocial neuropeptides and ANS function in WS

In this section, we attempt to consolidate the ANS data on WS with some relevant findings on OT and AVP. In the context of the broader literature on prosocial neuropeptides, the findings of elevated base line levels as well as peak release of OT and AVP to emotional stimulation in WS relative to TD (Dai et al., 2012) appear consistent with the social profile of WS that is associated with increased approach and proclivity toward engaging the eyes, as well as maladaptive behaviors. Importantly, Dai et al. (2012) reported a positive association between basal OT level and approach, and a negative correlation with adaptive social behaviors, for individuals with WS, suggesting that some aspects of the increased OT indeed are maladaptive. The finding linking intranasal OT administration to pupil dilation (Wiseman and Watt, 2010) appears surprising in light of the data of Plesa Skwerer et al. (2011) indicating reduced pupil dilation in WS, as perhaps the opposite could have been expected. Intranasal OT administration has also been suggested to be associated with increased HRV (Kemp et al., 2012a,b). Järvinen et al. (2012) reported decreased HRV within the visual domain, and increased HRV within the auditory domain, in individuals with WS, suggesting context-dependent or unstable HRV in WS. In the study of Dai et al. (2012), no significant associations between HR and blood pressure measures and neuropeptide function were observed, also suggesting a complex mechanism in WS. Future studies should thus establish HRV in WS in the resting state. Further, studies employing sensitive cardiac indices of ANS function in WS are acutely needed to clarify the inconsistencies in the current literature, and to allow the data to be linked to theories of social behavior. At the same time, the existing evidence may reflect some degree of heterogeneity in ANS function in WS, which may be further exacerbated by the fact that individuals with WS commonly present with hypertension and cardiac abnormalities (Pober, 2010), which may impact ANS function. In a similar vein, Dai et al. (2012) noted in their study that OT and AVP function was variable in their sample of individuals with WS.

## Determinants of social behavior: insights from WS

The picture of ANS function that is emerging from investigations of individuals with WS suggest that virtually in all studies, the typical elevation in arousal in response to (live) face stimuli in such individuals is present, despite the fact that baseline arousal levels may appear atypically low. This finding is typically seen in EDA-based analyses, while cardiac-based indices indicated hyperarousal to faces in WS (Järvinen et al., 2012). Thus, the evidence does not suggest hyporesponsivity to faces in WS *per se*. Further, individuals with WS were found to lack the typical habituation effect to face stimuli, suggesting that social information may retain its originality for those with the syndrome. Evidence further supported the uneven patterns of neural and behavioral responsivity across positive (preserved) vs. negative (compromised) social information (e.g., Haas et al., 2009) in WS, as such individuals demonstrated diminished arousal as indexed by pupil dilation to negative facial expressions (Plesa Skwerer et al., 2011), while within both visual and auditory social domains, increased HRV to happy stimuli was evident. This constellation of evidence fits in well with the social-behavioral characteristics of WS.

Future studies should determine the degree of heterogeneity within the WS population with respect to ANS function by testing sizeable sample of participants; this is crucial for being able to ultimately map social–emotional profiles in terms of behavior, and neural and hormonal characteristics, onto patterns of ANS function reliably. Contributing factors to some of the inconsistencies in the existing, scarce literature may include differences in experimental paradigms (ranging from arithmetic tasks to static/dynamic displays of affective faces), age ranges of participants, whether ANS activity was assessed using EDA vs. HR derived measures, and whether the effects of endogeneous vs. extrageneous OT were measured (cf. Churchland and Winkielman, 2012). Of the studies addressing ANS function in WS, only Järvinen et al. (2012) utilized indices of HRV, allowing more direct comparisons with the tenets of the polyvagal theory (Porges, 2007) and the social approach/avoidance hypothesis (Kemp and Guastella, 2011). Nevertheless, the evidence discussed in this article highlights that the study of ANS function in tandem with neuropeptide systems promises to open up an exciting avenue for the quest toward understanding the underpinnings of the social behavior of WS, including its positive as well as maladaptive features. Such studies may also prove helpful in identifying sensitive areas for intervention.

### Conflict of interest statement

The authors declare that the research was conducted in the absence of any commercial or financial relationships that could be construed as a potential conflict of interest.
